# Complete mitochondrial genomes of four deep-sea echinoids: conserved mitogenome organization and new insights into the phylogeny and evolution of Echinoidea

**DOI:** 10.7717/peerj.13730

**Published:** 2022-07-28

**Authors:** Shao’e Sun, Ning Xiao, Zhongli Sha

**Affiliations:** 1 Department of Marine Organism Taxonomy and Phylogeny, Institute of Oceanology, Chinese Academy of Sciences, Qingdao, China; 2 Laboratory for Marine Biology and Biotechnology, Qingdao National Laboratory for Marine Science and Technology, Qingdao, China; 3 Shandong Province Key Laboratory of Experimental Marine Biology, Institute of Oceanology, Chinese Academy of Sciences, Qingdao, China; 4 College of Biological Sciences, University of Chinese Academy of Sciences, Beijing, China

**Keywords:** Echinoidea, Deep-sea, Mitochondrial genome, Phylogenetic relationship, Evolution

## Abstract

Echinoids are an important component in benthic marine environments, which occur at all depths from the shallow-water hard substrates to abyssal depths. To date, the phylogeny of the sea urchins and the macro-evolutionary processes of deep-sea and shallow water groups have not yet been fully resolved. In the present study, we sequenced the complete mitochondrial genomes (mitogenomes) of four deep-sea sea urchins (Echinoidea), which were the first representatives of the orders Aspidodiadematoida, Pedinoida and Echinothurioida, respectively. The gene content and arrangement were highly conserved in echinoid mitogenomes. The *tRNA-Ser*^AGY^ with DHU arm was detected in the newly sequenced echinoid mitogenomes, representing an ancestral structure of *tRNA-Ser*^AGY^. No difference was found between deep-sea and shallow water groups in terms of base composition and codon usage. The phylogenetic analysis showed that all the orders except Spatangoida were monophyletic. The basal position of Cidaroida was supported. The closest relationship of Scutelloida and Echinolampadoida was confirmed. Our phylogenetic analysis shed new light on the position of Arbacioida, which supported that Arbacioida was most related with the irregular sea urchins instead of Stomopneustoida. The position Aspidodiadematoida (((Aspidodiadematoida + Pedinoida) + Echinothurioida) + Diadematoida) revealed by mitogenomic data discredited the hypothesis based on morphological evidences. The macro-evolutionary pattern revealed no simple onshore-offshore or an opposite hypothesis. But the basal position of the deep-sea lineages indicated the important role of deep sea in generating the current diversity of the class Echinoidea.

## Introduction

The sea urchin (Echinoidea) is one of the most iconic lineages of marine animals, representing a numerically relatively small class (slightly over 1,000) of marine invertebrates (Kroh & Mooi, 2022). The small number of extant echinoid species is opposed by its outstanding fossil record, with more than 10,000 nominal fossil species (Kier & Lawson, 1978; Kroh, 2010). Echinoids are an important component in benthic marine environments, and they occur at all depths from the shallow-water hard substrates to abyssal depths (Emlet, 2002; Furman & Heck, 2009; Tuya et al., 2004; Schultz, 2015). All extant 15 echinoid orders, except Stomopneustoida, have deep-sea representatives including 350 species (Glover, Higgs & Horton, 2022). The sea urchins have a long independent evolutionary history, which split from their closest relatives (the sea cucumbers) at Ordovician, more than 450 million years ago (Mya) (Smith, 1984; Smith & Savill, 2001; Thompson et al., 2017).

**Figure 1:**
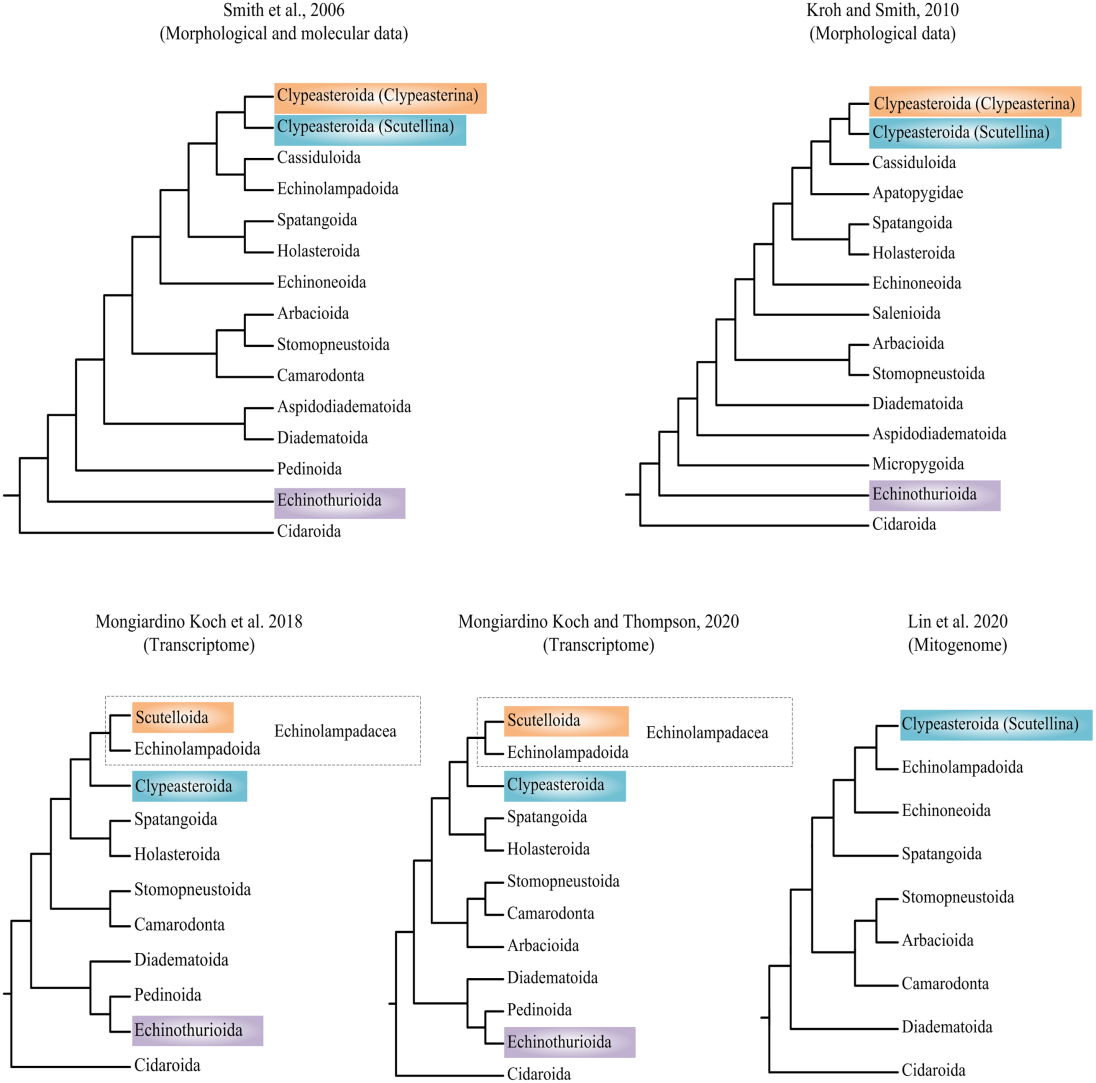
Recent hypotheses of relationships at orders of Echinoidea based on molecular and morphological data. The studies summarized here reached different conclusions.

Researchers have attempted to explore the phylogeny of Echinoidea based on morphological and molecular data (summarized in Fig. 1) (Smith et al., 2006; Kroh & Smith, 2010; Mongiardino Koch et al., 2018; Lin et al., 2020; Mongiardino Koch & Thompson, 2020). Phylogenetic incongruences between morphological and molecular data have been found, which are mainly concentrated on the positions of orders Echinothurioida and Clypeasteroida. The morphological evidences placed Echinothurioida as the sister clade to all other Euechinoidea (Smith et al., 2006; Kroh & Smith, 2010). The molecular evidences from transcriptomes, however, revealed a derived position of Echinothurioida, nested in a group that contains orders Diadematoida and Pedinoida (Mongiardino Koch et al., 2018; Mongiardino Koch & Thompson, 2020). The phylogenetic placement of Clypeasteroida and Echinolampadoida is another important issue in echinoid phylogeny. The suborders Clypeasterina and Scutellina were once placed in the order Clypeasteroida based on the morphological characters (Smith et al., 2006; Kroh & Smith, 2010). Based on the morphological evidences, Clypeasteroida was a monophyletic sister to Cassiduloida (Kroh & Smith, 2010) or Cassiduloida/Echinolampadoida lineages (Smith et al., 2006). Mongiardino Koch et al. (2018) modified Scutellina and Echinolampadoida to suborder level and placed them in the order Echinolampadacea based on the transcriptomic data. In their study, Scutellina and Clypeasterina were renamed to Scutelloida and Clypeasteroida, respectively. The transcriptomic study further suggested a close relationship between Clypeasteroida and Echinolampadacea (Mongiardino Koch et al., 2018; Mongiardino Koch & Thompson, 2020). The recent phylomitogenomic analyses failed to sample the taxa necessary to test these hypothesis (Lin et al., 2020). Therefore, the origin and evolution research of sea urchin is now limited by the topological uncertainty.

Few attempts have been made to address the relationship between the shallow-water and deep-sea lineages of Echinoids (Smith & Stockley, 2005). Deep sea (greater than 200 m) occupies about 66% of the bottom of global ocean, representing the largest and most remote biome of the world (Woolley et al., 2016). It has special environment factors, such as low temperatures, low oxygen level, high hydrostatic pressure, limited food and constant darkness (Naganoa & Nagahama, 2012). Deep sea plays an important part in generating the current patterns of marine biodiversity (Carney, 2005; Brown & Thatje, 2014). Explaining the origin of deep-sea organisms is still a central priority for marine biogeographic research (McClain & Hardy, 2010). Evidences now suggest that most faunal groups may have originated in shallow water (Strugnell et al., 2008; Raupach et al., 2009; Yang et al., 2015; Sun, Sha & Wang, 2019). Despite this, it is assumed that evolutionary innovations in stylasterid corals are generated in deep sea (Lindner, Cairns & Cunningham, 2008). The evolution of echinoderms seems more complicated. The phylogenomic analysis of ophiuroids suggested that most of the oldest lineages showed a deep-sea origin, however, the multiple colonization events indicated that the evolution of brittle stars were neither a simple onshore-offshore pattern, nor the opposite hypothesis (Thuy, 2013; Bribiesca-Contreras et al., 2017). The phylomitogenetic relationship of the sea cucumbers showed that the deep-sea species formed the basal clades (Sun, Sha & Xiao, 2021). Thus, the role of the deep sea in biogeographic processes cannot be ignored.

The mitochondrial genome (mitogenome) is characterized by several advantages, such as maternal inheritance, small genome size, fast evolutionary rates and low recombination (Boore, 1999; Curole & Kocher, 1999). Furthermore, the mitogenome can provide more phylogenetic signals than single mtDNA markers, therefore, it is widely used for phylogenetic and evolutionary analysis at different taxonomic level of the Echinoderma (Perseke et al., 2010; Reich et al., 2015; Láruson, 2017; Bronstein & Kroh, 2019; Galaska et al., 2019). Prior to our study, only thirty-nine mitogenomes from Echinoidea are available, which distributed within seven orders (Cidaroida, Diadematoida, Arbacioida, Camarodonta, Stomopneustoida, Clypeasteroida, Spatangoida), with nine orders still no representatives. Compared to their diversity and abundance, the mitogenomes available are limited in the class Echinoidea, and only four mitogenomes of deep-sea species have been reported (Kober & Bernardi, 2013; Direct submission for MW354512). Inadequate taxon sampling and taxon biases can lead to topological distortions owing to the artefactual sources of error (Timm & Bracken-Grissom, 2015). Thus, improving the representation of mitogenomes for Echinoidea is indispensable for understanding the phylogenetic and evolution of sea urchin.

We newly sequenced four complete mitogenomes of the deep-sea sea urchins (*Aspidodiadema arcitum* Mortensen, 1939, *Caenopedina pulchella* (Agassiz & Clark, 1907), *Phormosoma bursarium* Agassiz, 1881, *Araeosoma owstoni* Mortensen, 1904, which were the first representatives of the orders Aspidodiadematoida, Pedinoida and Echinothurioida, respectively. In this study, we aim to improve our understanding of Echinoidea phylogeny and evolution of deep-sea sea urchins by: (1) comparing the organization and composition of deep-sea echinoid mitogenomes with those of shallow water ones; (2) conducting a phylogenetic investigation on Echinoidea with expanded mitogenome data and taxon sampling; (3) exploring the macro-evolutionary processes of deep-sea and shallow water echinoids.

## Materials and Methods

### Specimen collection

Adults of these four sea urchins were originally collected from the Tropical western Pacific. The collection information was shown in Table 1. All samples were preserved in 95% ethanol immediately following collection and stored at Marine Biological Museum, Chinese Academy of Sciences, Qingdao, China before DNA extraction.

**Table 1:** New mitochondrial genomes analyzed in the present study.

Pecies	*Aspidodiadema arcitum*	*Phormosoma bursarium*	*Caenopedina pulchella*	*Araeosoma owstoni*
Genbank Accession No.	ON254173	ON254174	ON254175	ON254176
Mitogenome size (bp)	15,870	15,747	15,745	15,704
Clean reads	38,158,676	14,680,228	35,586,080	39,932,402
Coverage depth	104x	77x	118x	26x
Location	Tropical Western Pacific Ocean	Tropical Western Pacific Ocean	Tropical Western Pacific Ocean	Tropical Western Pacific Ocean
Positioning	FX-Dive172	FX-Dive63	FX-Dive14	FX-Dive 214
Coordinates	17°23′24.206″N	11°16′30.831″N	8°51.6314372′N	10°05′24″N
	153°05′34.892″E	139°22′10.598″E	137°47.1799584′E	140°10′03″E
Depth (m)	1355	1488.4	288	1497
Cruise details	Remotely Operated Vehicle (ROV)	Remotely Operated Vehicle (ROV)	Remotely Operated Vehicle (ROV)	Remotely Operated Vehicle (ROV)
Collect time	2018.4.1	2016.3.21	2014.12.12	2019.6.1

### Mitochondrial genome sequencing and assembly

Total genomic DNA was extracted from each Echinoidea species with E.Z.N.A® Tissue DNA kit (OMEGA, Wuhan, China) according to the manufacture’s instructions. The paired-end libraries were obtained using TruSeq™ Nano DNA Sample Prep Kit (Illumina, San Diego, CA, USA) with an average insert size of 450 bp. Subsequently, Illumina (San Diego, CA, USA) HiSeq 4000 platform was used to sequence 2 × 150 bp paired-end reads. The clean data were obtained from each library after trimming using Trimmomatic (Bolger, Lohse & Usadel, 2014) with the following parameters: ILLUMINACLIP:TruSeq3-PE.fa:2:30:10 LEADING:3 TRAILING:3 SLIDINGWINDOW:4:15 MINLEN:75. The clean sequences (Table 1) were then assembled using SPAdes v3.10.1 (k-mer = 21–77) (http://bioinf.spbau.ru/spades) and NOVOPlasty software (Dierckxsens, Mardulyn & Smits, 2017) with default parameters. BLAST (Altschul et al., 1997) was conducted to identify the contigs of mitochondrial origin. In the seed extension algorithm of NOVOPlasty, the *cox1* or 16S rDNA gene fragments of their closed related species were used as seed sequences (16S of *A. jacobi* (DQ073734) for *A. arcitum*, *cox1* of *C. novaezealandiae* (JN259400) for *C. pulchella*, *cox1* of *Araeosoma* sp. (MN868991) for *P. bursarium* and *A. owstoni*). NOVOPlasty will circularize the mitogenomes when the length is in the expected range and both ends overlap by at least 200 bp.

### Gene annotation and sequence analysis

The four mitogenomes were initially annotated by MITOS web server (Bernt et al., 2013) with invertebrate genetic codes. The protein coding genes (PCGs) boundaries were further annotated using the Open Reading Frame Finder (ORF Finder) (https://www.ncbi.nlm.nih.gov/orffinder/). The ribosomal RNA (rRNA) genes were edited by alignment with homologous genes of closely related species. The tRNA genes and their secondary structures were further predicted with ARWEN 1.2.3.c (Laslett & Canbäck, 2007). The newly sequenced mitogenomes can be accessed from the GenBank database with the accession numbers ON254173–ON254176 (Table S1).

The nucleotide composition and codon usage were calculated with MEGA 6.0 (Tamura et al., 2013) based on the invertebrate mitochondrial genetic code (genetic code = 5). The bias of the nucleotide composition was measured by AT and GC skews: AT skew = (A − T)/(A + T) and GC skew = (G − C)/(G + C) (Perna & Kocher, 1995). Non-synonymous to synonymous substitution rates (Ka/Ks, *ω*) were calculated by PAML package in CODEML program (Xu & Yang, 2013). Comparisons between the deep-sea and shallow water echinoid groups were performed by the chi-square test in IBM SPSS Statistics, release 19.0.0.1 to the nucleotide compositions, codon usage counts and Ka/Ks rates.

### Phylogenetic analyses

The mitogenomic phylogenetic trees were reconstructed with 54 echinoid species, including the four newly determined mitogenomes, and 11 sequences extracted from transcriptomes (Table S2). Three Asteroidea species (*Echinaster brasiliensis* (MG636999), *Asterias amurensis* (AB183559), *Culcita novaeguineae* (MT476594)) were used as outgroups.

The saturation of substitution was evaluated using DAMBE6 (Xia, 2017), by plotting the number of transitions (s) and transversions (v) *versus* the F84 genetic distance (Felsenstein, 1984), and comparing the information entropy-based index (I_ss_) with critical values (I_ss.c_) (Xia et al., 2003; Xia & Lemey, 2009). If I_ss_ was significantly lower than I_ss.c_, the sequences were considered little substitution saturation.

In this study, the phylogenetic analyses were conducted by two datasets, one with 12 PCGs (except atp8 since a high saturation was detected on this gene) nucleotide sequences at the first and second codon positions (without the 3rd codon since a high saturation was detected on this position), hereafter referred to as the NT data set; one with 13 PCGs amino acid sequences, hereafter referred to as the AA data set. The nucleotide sequences and amino acid sequences of 12 PCGs were aligned separately using MEGA 6.0 (Tamura et al., 2013). The codon alignment algorithm was used for the alignment of nucleotide sequences. The alignments of 12 individual PCGs were then concatenated into a single supermatrix using FASconCAT (Kück & Meusemann, 2010). PartitionFinder 2 and PartitionFinder Protein 2 (Lanfear et al., 2017) were used to select the best-fit substitution model of nucleotide and amino acid substitution (Table S3). The phylogenetic analyses of Maximum likelihood (ML) and Bayesian inference (BI) were conducted for each dataset. ML analysis was performed in IQ-TREE web server (Trifinopoulos et al., 2016) with the best-fit partition schemes and models. The node reliability was assessed using 5,000 ultrafast bootstrap replicates (Minh, Nguyen & Von Haeseler, 2013). The BI analysis was conducted using MrBayes 3.1 software (Ronquist & Huelsenbeck, 2003) with partition models. The Markov chain Monte Carlo (MCMC) runs of 10,000,000 generations were conducted, and trees were sampled every 1,000 generations with a burn-in of 25%. The software Tracer v1.7 (Rambaut et al., 2018) was used to check the parameters (effective sampling size for all parameters > 200). All runs reached convergence (average standard deviation of split frequencies decreased to <0.01 and remained stable).

### Ancestral habitat reconstruction

The ancestral habitat of the echinoids was reconstructed using three different models, *i.e.,* Statistical Dispersal-Vicariance Analysis (S-DIVA), Statistical Dispersal-Extinction-Cladogenesis (S-DEC) and Dispersal-Extinction-Cladogenesis (DEC) implemented in RASP v. 3.2 (Yu et al., 2015). Distribution data of the sea urchins were gathered from Ocean Biodiversity Information System (https://obis.org/). A distinction was made between shallow (0–200 m) and deep sea (below 200 m) (Thistle, 2003). According to Bribiesca-Contreras et al. (2017), the criteria for assigning a species into shallow water (S) or deep-sea (D) was that at least 10% of the total occurrence records were collected from this depth bathome. For the intermediate and eurybathic distributions, a both bathomes (B) was assigned for the species.

## Results

### Mitogenome organization

**Figure 2:**
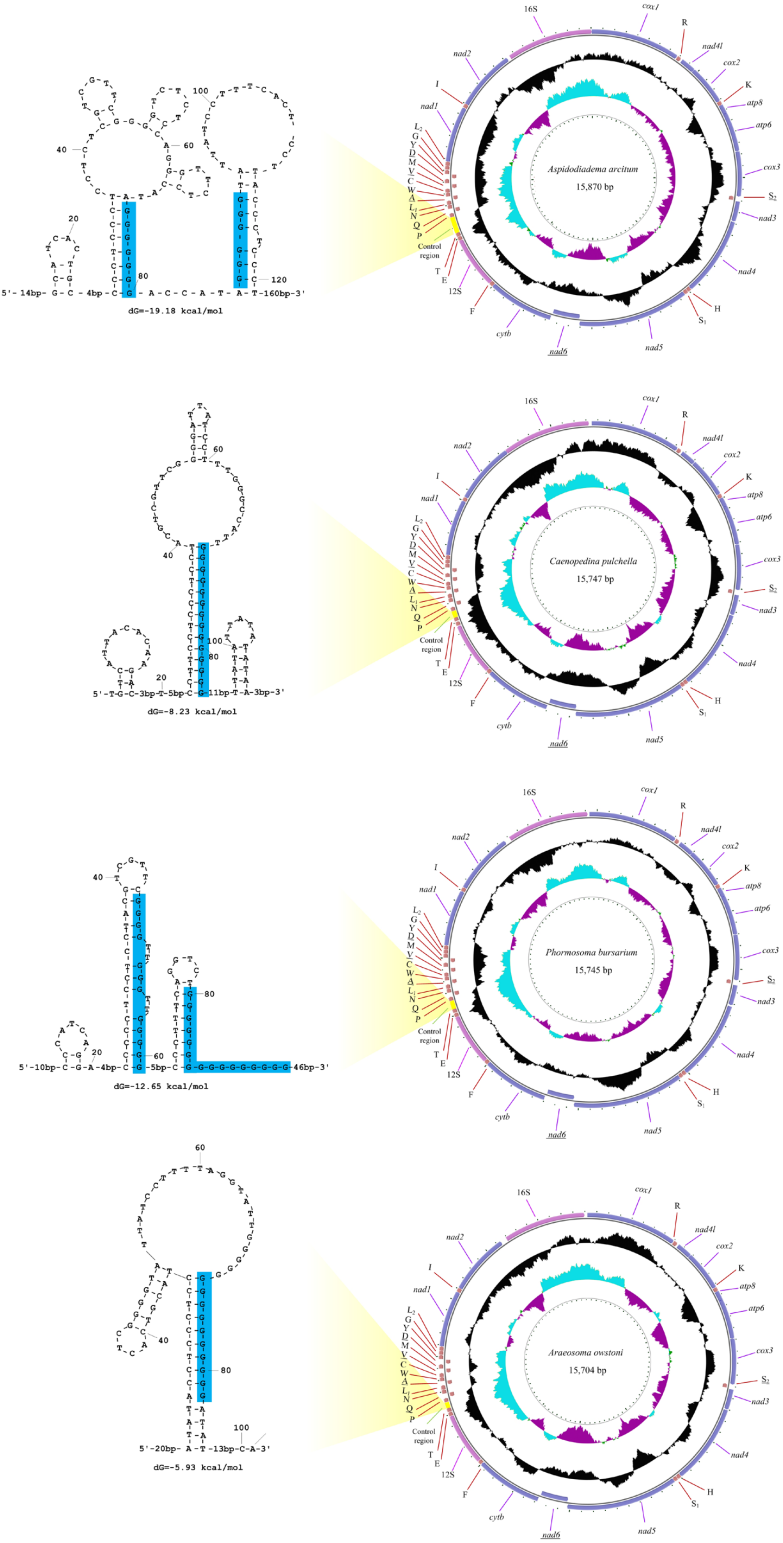
The organization of the mitochondrial genome of the four newly determined mitogenomes. The full names of protein-coding genes and rRNA genes are listed under abbreviations. One uppercase letter amino acid abbreviations are used to label the corresponding tRNA genes. The position of control region (CR) is marked in green, and their stem-loop structures are shown in left. The poly-G stretches are indicated in blue.

The sequencing output data for the four deep-sea sea urchins were summarized in Table 1. The mitogenomes of the four echinoids were all circularized from the reads, with 15,704 (*A. owstoni*) to 15,870 bp (*A. arcitum*) in length. The average coverage were 26–104x for these four mitogenomes. These new mitogenomes contained 13 protein-coding genes (PCGs), 2 ribosomal RNA genes (rRNAs), 22 transfer RNA genes (tRNAs) and a control region (CR) (Fig. 2, Table S4). Among the 37 genes, six (*nad6*, tRNA-Ser^UCN^, tRNA-Gln, tRNA-Ala, tRNA-Val, tRNA-Asp) located on the heavy-strand (H-strand), with the remaining ones on the light-strand (L-strand). The gene arrangement of the four new mitogenomes were identical to that of other echinoids.

**Figure 3:**
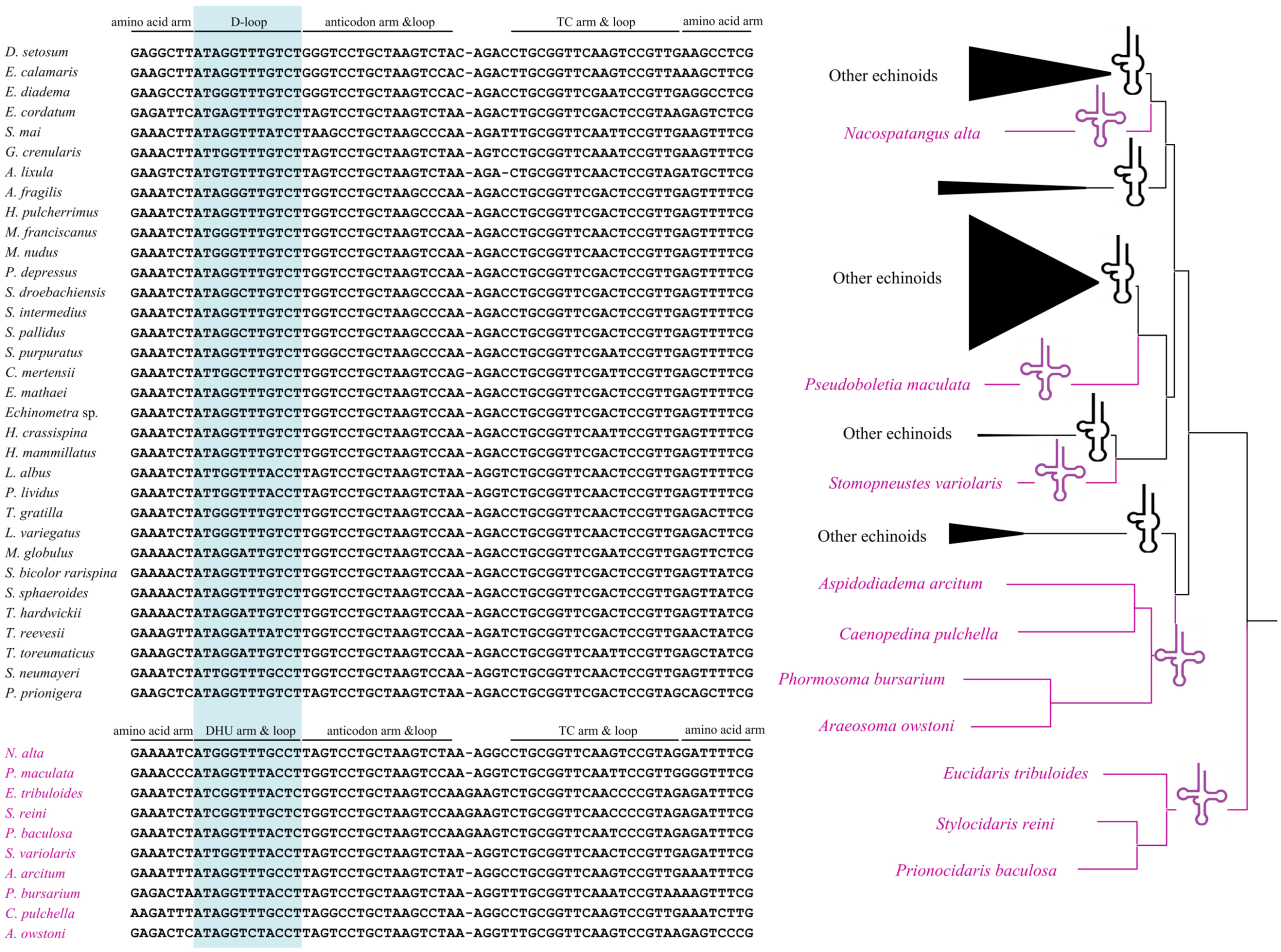
A possible evolutionary pathway of tRNA-Ser^AGY^ gene and secondary structure changes (loss and acquisition of the DHU arms) among mitochondrial genomes of the echinoids.

All the tRNA genes can be folded into canonical clover-leaf secondary structures. Unlike most of the echinoids, the tRNA-Ser^AGY^ gene showed complete DHU arm (hereafter D-arm) and loop (hereafter D-loop). In order to understand the evolution of tRNA-Ser^AGY^ gene among the echinoids, we mapped the structures of tRNA-Ser^AGY^ on the phylogenetic tree of echinoids constructed in this study (Fig. 3). Under this framework, we found the normal cloverleaf pattern was the most basal and was maintained in only several lineages, while the D-arm lost in most species.

The 12S and 16S rRNA genes exhibited similar size among the four new genomes (895–906 bp and 1,415–1,520 bp, respectively). Compared with the other regions, the control regions (CRs) of the four mitogenomes exhibited more variation in length, ranging from 101 bp in *A. owstoni* to 281 bp in *A. arcitum*. Obviously, the length variation among them were congruent with the differences of their mitogenome sizes. The echinoid CRs harbored a poly-G stretch, which participated in the formation of the stem-loop structures (Fig. 2).

### Base composition and codon usage

**Figure 4:**
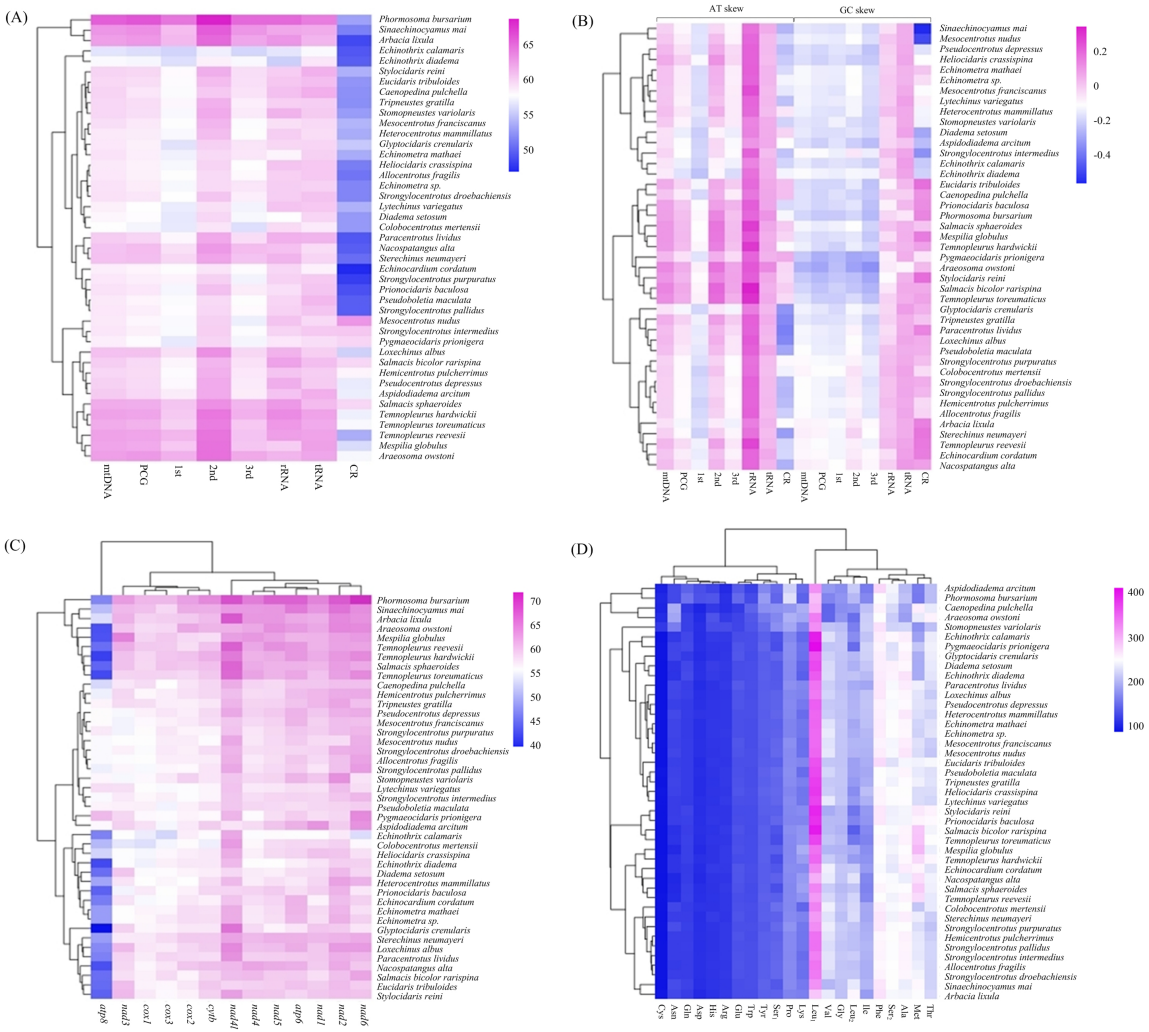
(A–D) Nucleotide composition and codon usage of of Echinoidea mitogenomes.

The overall A + T content of the four new mitogenomes ranged from 59.94% in *C. pulchella* to 65.68% in *P. bursarium* (Fig. 4A, Table S5). The values of the AT-skews were mostly positive, indicating more As than Ts. And the GC-skews for all echinoid mitogenomes were positive, suggesting Gs were more abundant than Cs (Fig. 4B, Table S5). tRNA and rRNA showed the highest (60.60% and 60.58%, respectively) A + T content, while the CR showed the lowest (53.48%) A + T content. The nucleotide composition analysis of the PCGs suggested that *nad2*, *nad4l* and *nad6* possessed the highest A + T content (more than 60%), while that of the *atp8* gene was the lowest. Furthermore, the third codon position of the PCGs had higher A + T content (64.25%) than that of the first and second codon positions (54.19% and 60.03%, respectively) (Fig. 4C, Table S5).

Significantly, among the reported echinoid mitogenomes, the deep-sea species *P.bursarium* showed the highest A + T content in mtDNA, PCG, tRNA, and rRNA. In order to investigate if the base composition between the deep-sea and shallow-water species was different, the A + T content at complete mtDNA, PCG, tRNA, rRNA genes and CR were compared. However, statistical *t*-tests proved that only the difference of A + T content in tRNA between deep-sea and shallow water echinoids was significant (*p* = 0.047).

In the four newly sequenced mitogenomes, almost all PCGs (except *atp8*) started with the typical ATN. The typical stop codon TAA was used more frequently than TAG, while the incomplete stop codons T (*nad4* in *P. bursarium* and *A. owstoni*) and TA (*cytb* in *P. bursarium*) were also used as the stop codon. The codon usage analysis of the proteins (Fig. 4D, Table S5) indicated that Leu_1_, Phe, Met, Ser_2_, Thr and Ala were the six most common amino acids, accounting for about a half of the total. The relative synonymous codon usage (RSCU) further revealed a base composition bias in echinoid mitogenomes. Usually, the RSCU values of NNU and NNA were higher than 1 (Table S5).

### Phylogenetic relationships of Echinoidea

**Figure 5:**
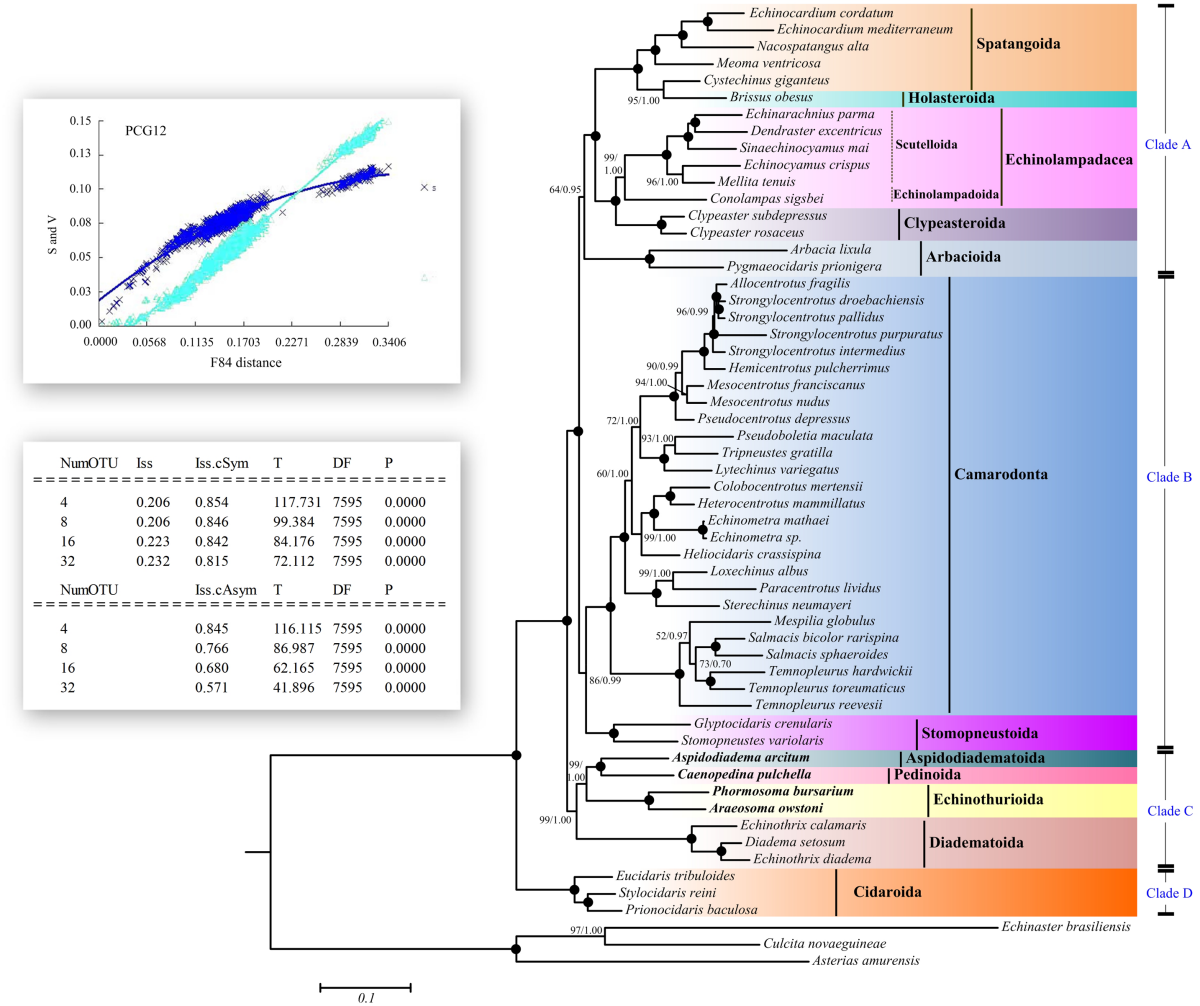
Saturation tests and phylogenetic trees inferred from ML and BI methods based the 12 concatenated mitochondrial genes (except *atp8* gene). The bootstrap probability (the first number) and the Bayesian posterior probability (the second number) were shown at each node. The black dots mean 100/1.00. The four echinoid species newly sequenced in the present study have been used bold taxa.

Saturation tests were performed for each of the PCG genes, the dataset PCG1 (the first codon position), PCG2 (the second position) and PCG3 (the third position). Substitution saturation was detected in *atp8* gene as reflected from the linear correlation of the number of transitions (s) and transversions (v) plotted against the F84 genetic distance, as well as from a significantly higher value of I_ss_ in comparison to I_ss.c_ (Fig. 5, Table S6, Fig. S1). The dataset PCG3 exhibited some saturation (Fig. S1), although I_ss_ was significantly lower than I_ss.c_ (Table S6).

**Figure 6:**
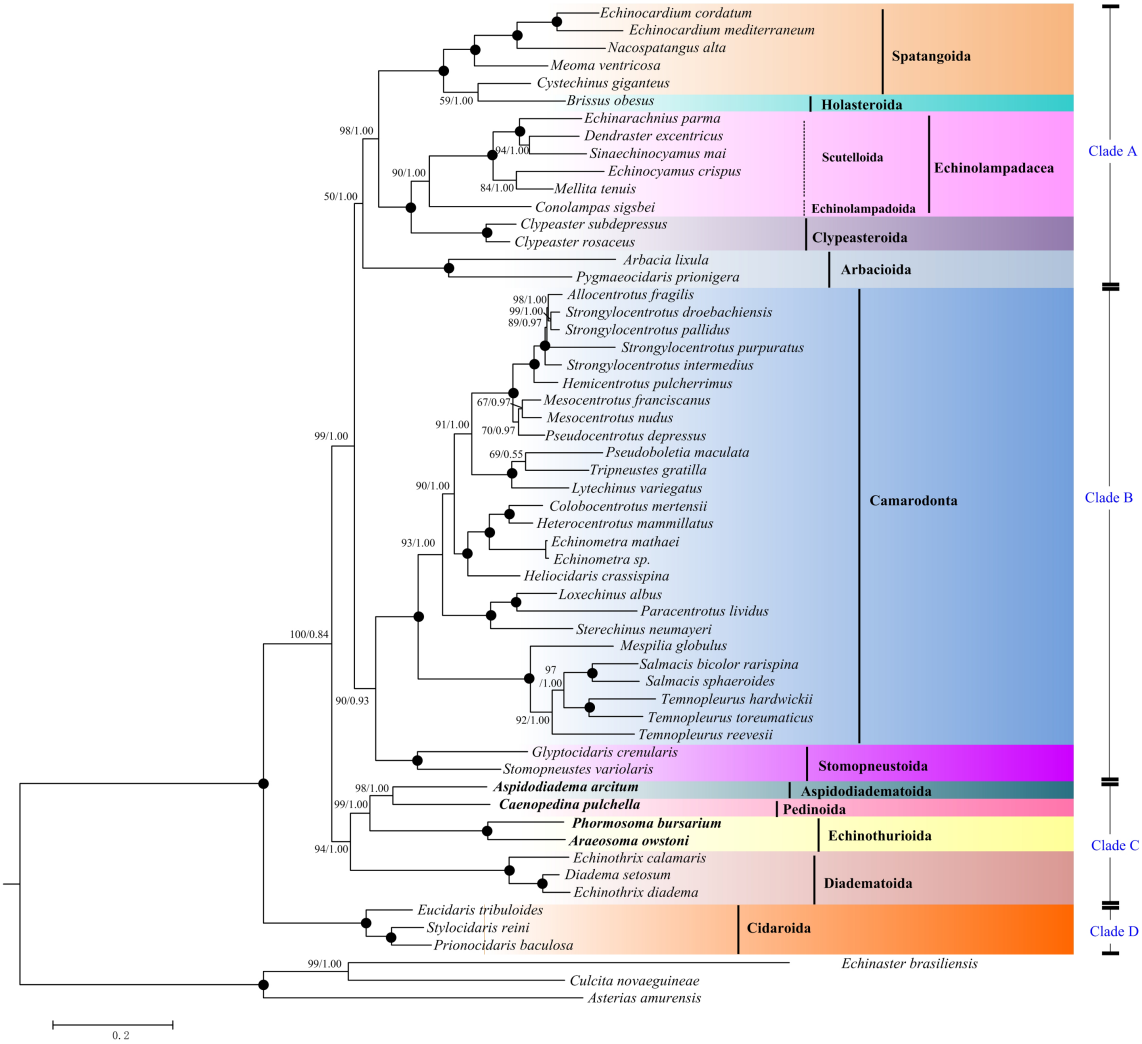
Phylogenetic trees (ML and BI) based on the amino acid sequences of the 13 mitochondrial genes. The bootstrap probability (the first number) and the Bayesian posterior probability (the second number) were shown at each node. The black dots mean 100/1.00. The four echinoid species newly sequenced in the present study have been used bold taxa.

Maximum Likelihood (ML) and Bayesian inference (BI) trees based on the nucleotide sequences (NT) (Fig. 5) and amino acid sequences (AA) (Fig. 6) datasets were mostly congruent. The tree topologies resulting from the BI and ML analysis were identical. The phylogenetic trees supported the monophyly of all the echinoid orders except Spatangoida. *Cystechinus giganteus* (Holasteroida) appeared as subgroup within, rather than a sister group to Spatangoida. In both topologies, Echinoidea was divided into four groups (Clades A–D in Fig. 5). The order Cidaroida (Clade D) was placed as a basal sister group to the clades of the remaining echinoids. Arbacioida and the irregular sea urchins (Spatangoida + Holasteroida + Echinolampadacea + Clypeasteroida) clustered together (Clade A). Camarodonta showed the closest relationship with Stomopneustoida (Clade B), and then clustered together with Clade A. Aspidodiadematoida, Pedinoida, Echinothurioida and Diadematoida formed a sister clade (Clade C), which was supported as the sister clade to Clade A + Clade B.

### Habitat origin of the echinoids

**Figure 7:**
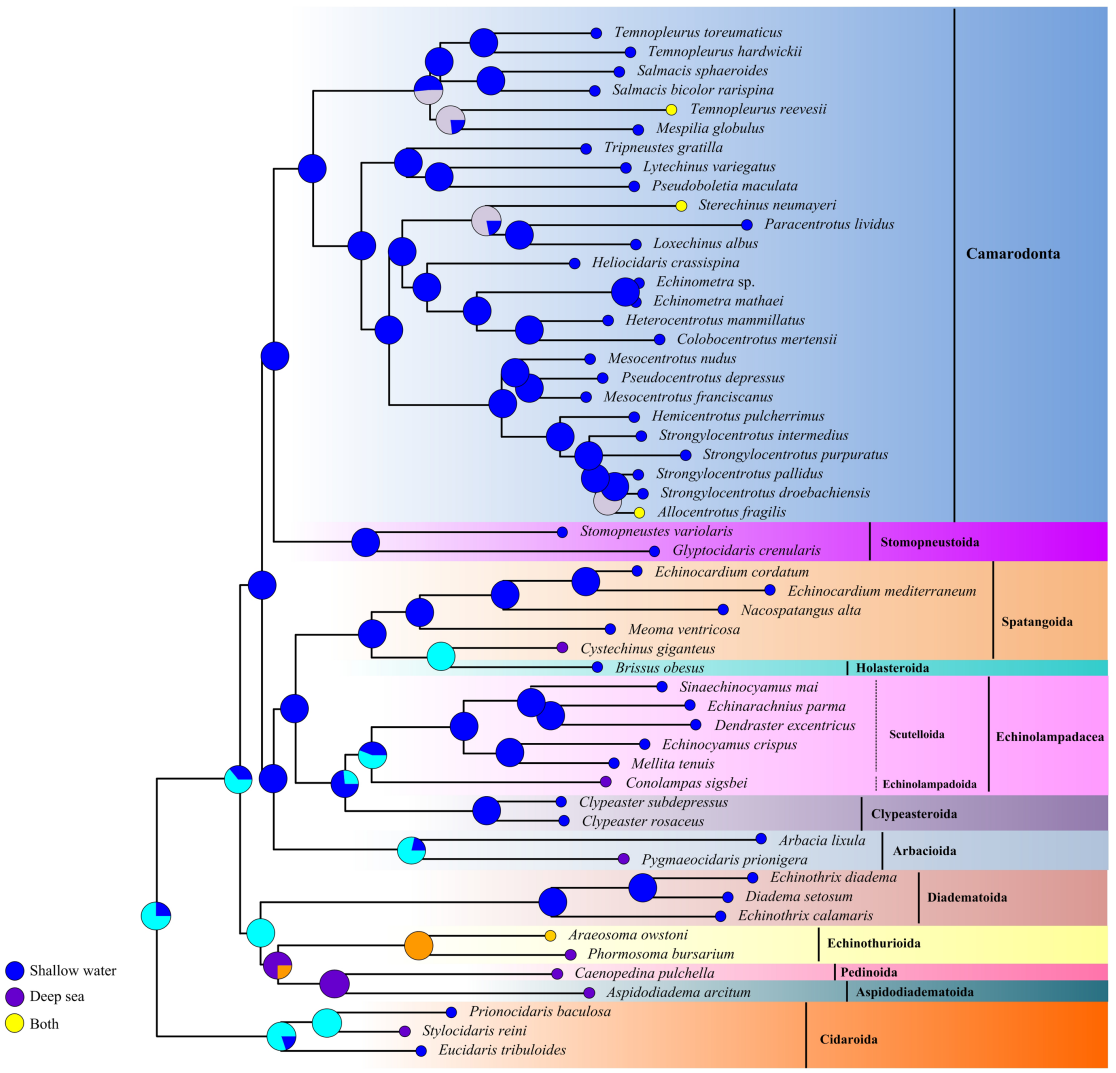
Historical habitat of Echinoidea. Pie charts near nodes indicated the probabilities of certain ancestral habitat. Red clades indicated the deep-sea species.

The ancestral habitat reconstruction provides new insights into the origin of echinoids (Fig. 7). Our result did not support a consistent offshore to onshore or onshore to offshore pattern across the echinoid phylogeny, but multiple transitions between deep sea and shallow water lineages supporting a mixed origin. Although the deep-sea echinoid species were slightly underrepresented in this study, they were recovered as older lineages, indicating that the deep-sea habitats might be an important site for origination.

## Discussion

In this study, we successfully sequenced and analyzed four complete mitochondrial genomes of deep-sea Echinoidea species. They are the first representatives of the orders Aspidodiadematoida, Pedinoida and Echinothurioida. Compared with the lengths of other previously sequenced echinoid sequences (15,650 bp in *Strongylocentrotus purpuratus* to 16,358 bp in *Pygmaeocidaris prionigera*), the sequence of *A. arcitum* (15,870 bp) obtained here is the second largest one. The minor length differences of all the PCGs (<20 bp, and *cox2*, *cox3*, *cytb* and *nad4l* are of the exact same length) indicates that the PCGs have relatively conserved features. The location and length of tRNAs are highly conserved. Compared with PCGs and tRNAs, the length of rRNAs show relatively high variations (2,402 bp in *Temnopleurus reevesii* to 2,504 bp in *Stylocidaris reini*). The differences in mitogenome size of echinoids are mainly attributed by the variations of the CR (101 bp in *A. owstoni* to 677 bp in *P. prionigera*).

No gene rearrangements are found in Echinoidea. Indeed, the gene order and orientation of the mitogenomes of the echinoids are identical to the echinoderm ground pattern (Shen et al., 2009). Among the Echinodermata, the genome architecture of Echinoidea is most similar to that of the vertebrate basal genome differing only in the transpositions of the 16S rRNA and the *nad4l* gene (Boore, 1999).

Among the 22 putative tRNA secondary structures in metazoans, tRNA-Ser^AGY^ usually lacks a DHU stem and therefore forms a simple loop structure (Lavrov, Brown & Boore, 2000; Ruiz-Pesini & Wallace, 2006). In contrast, the cloverleaf structure of tRNA-Ser^AGY^ has been found in ten echinoids, and most of the species with the structure locate in the basal lineages. We speculate that the tRNA-Ser^AGY^ with normal cloverleaf pattern represents an ancestral state. The derived D-loop structures of tRNA-Ser^AGY^ occurs in younger lineages. Previous studies have suggested that tRNA isomerisms really exist and are functional (Sakurai et al., 2006), and however whether the sporadically observed tRNA isomerism is related to adaptive processes awaits further investigation.

The calculation of A + T content in the echinoid mitogenomes reveals a A + T biased nucleotide composition. Among the partitions of mitogenome, the CR shows the lowest A + T content. This is contrast to the widely accepted phenomenon that the CR (also called AT-rich region) contains the highest AT content of the complete mitogenome (Zhang, Szymura & Hewitt, 1995). Several studies have found that in other echinoderms, *e.g.*, holothuroids (Sun, Sha & Xiao, 2021) and asteroids (Sun, Xiao & Sha, 2022), the A + T content of mtDNA, PCG, tRNA and and rRNA of deep-sea groups are significantly higher than those of the shallow-water species. However, this phenomenon has not been detected in the echinoids.

The RSCU values of echinoid mitogenomes indicate a strong A+T-bias in the third codon position of PCGs. This result is also consistent with the hypothesis that the codon usage bias may be positively correlated with the A + T-bias of the third codon position in mitogenomes (Salvato et al., 2008; Kim et al., 2009; Chai, Du & Zhai, 2012; Hao et al., 2012). However, unlike holothuroids (Sun, Sha & Xiao, 2021) and asteroids (Sun, Xiao & Sha, 2022), no difference has been found in the codon usage between the deep-sea and shallow water groups of echinoid.

In this study, a total of 12 representative orders of the Echinoidea have been selected for phylogenetic analysis to understand the genetic relationship among each order. The topology based on mitogenomes in this study is inconsistent with previous morphological and molecular studies (Smith et al., 2006; Kroh & Smith, 2010; Mongiardino Koch et al., 2018; Lin et al., 2020; Mongiardino Koch & Thompson, 2020). The closest relationship of Holasteroida and Spatangoida has been proved with morphological and transcriptomic dataset (Smith et al., 2006; Kroh & Smith, 2010; Mongiardino Koch et al., 2018; Mongiardino Koch & Thompson, 2020). However, the monophyly of Spatangoida is not supported in our result, with the lineage of Holasteroida nested as a subgroup, instead of the sister group of Spatangoida. Recent transcriptomic studies have highlighted the incongruence between molecular trees and morphological trees related to the position of Scutelloida and Clypeasteroida (Mongiardino Koch et al., 2018; Mongiardino Koch & Thompson, 2020). Our mitogenomic phylogenetic work confirms the closest relationship between Scutelloida and Echinolampadoida, and supports the hypothesis of Mongiardino Koch et al. (2018) that Scutelloida and Echinolampadoida should be placed in order Echinolampadacea. The closest relationship of Arbacioida and Stomopneustoida has been recovered by the morphological and molecular evidences (Smith et al., 2006; Kroh & Smith, 2010; Lin et al., 2020; Mongiardino Koch & Thompson, 2020). Our investigation expands the representatives of Arbacioida and Stomopneustoida. We sheds new light on the position of Arbacioida, which indicates a novel sister relationship between Arbacioida and the irregular sea urchins. Another noteworthy result of our analyses is that we have first positioned Aspidodiadematoida in the phylogeny of Echinoidea using mitogenomic data. The relationship (((Aspidodiadematoida + Pedinoida) + Echinothurioida) + Diadematoida) discredits the hypothesis based on morphological evidences (Smith et al., 2006; Kroh & Smith, 2010). Inconsistency between molecular and morphological data has been found in many extant echinoderms (Janies, 2001; Miller et al., 2017). The incongruences demonstrate that further taxonomic revision is required for these groups.

There have been two contrasting hypothesises about the origin of deep-sea organisms. Some researchers have regarded them to be ancient (Wilson, 1999), while otherssupport their recent origin (Jacobs & Lindberg, 1998). The explanation of ancient origin has been evidenced by the discovery of relict species from the deep sea (Gage & Tyler, 1999), indicating that some modern deep-sea groups experienced situ diversification over evolutionary time (Lindner, Cairns & Cunningham, 2008). Furthermore, the geological history has recorded the extreme changes in oxygen, temperature and circulation, which may result in mass extinction of the deep-sea fauna (Jacobs & Lindberg, 1998; McClain & Hardy, 2010) followed by reinvasions of shallow-water organisms, *e.g.*, molluscans and crustaceans (Strugnell et al., 2008; Raupach et al., 2009; Sun, Sha & Wang, 2019). It seems that increasing evidences point to the important role of the shallow water in macro-evolutionary processes of marine faunas and that of the deep sea has been discounted or ignored. Our data does not show a consistent offshore to onshore or an opposite pattern across the echinoid phylogeny, as there have been multiple transitions back and forth between deep-sea and shallow-water lineages. However, the older lineages are suggested to have inhabited deep sea, indicating the important role of the deep sea in the diversification of Echinoidea. It has been reported that the aspidodiadematoids and echinothurioids sea urchins first appeared in Jurassic shallow-water environments, then disappeared from the fossil record, and survived in deep-sea until today (Smith & Stockley, 2005). In addition, the similar evolutionary pattern has also been found in other echinoderms, *e.g.*, holothuroids (Sun, Sha & Xiao, 2021), asteroids (Sun, Xiao & Sha, 2022) and ophiuroids (Bribiesca-Contreras et al., 2017). Perhaps this is not surprising. Previous studies have demonstrated that oceanic anoxic events did not have a strong effect on echinoderm deep-sea diversity (Smith & Stockley, 2005; Thuy et al., 2012). Diversification of primary producers and increasing nitrification of the deep sea environment may increase the diversity of deep-sea sea urchin (Smith, 2013) so they can escape from deep-sea hypoxia events and persist in the deep sea up to date. Based on this hypothesis, we can interpret why older echinoid clades have a deep-sea origin.

## Conclusion

In this study, we contributed four complete mitogenomes of deep-sea sea urchins from three orders, enhancing the taxonomic coverage of Echinoidea mitogenomic data. We did the first comparative analysis of mitogenome base composition, phylogenetic relationships and evolutionary history in Echinoidea. The genome architecture of Echinoidea was highly conserved. We identified the cloverleaf structure of tRNA-Ser^AGY^, previously considered missing DHU arm, and speculated that tRNA-Ser^AGY^ with DHU arm may represent the ancestral status. The base composition and codon usage showed no difference between deep-sea and shallow water groups. The phylogenetic relationships based on PCGs supported the monophyly of all the echinoid orders except Spatangoida. Cidaroida was placed as a basal sister group to all the others. We shed new light on the positions of Arbacioida and Aspidodiadematoida. Although the echinoid phylogeny showed no consistent offshore-onshore or onshore-offshore pattern, the deep-sea lineages positioned in the older clades revealed the important role of the deep sea in the diversification of Echinoidea.

## Supplemental Information

10.7717/peerj.13730/supp-1 Figure S1 Substitution saturation plot of the 13 PCGs The number of transitions (s) and transversions (v) is plotted against the F84 genetic distance. A linear correlation is sustained for both transitions and transversions as expected in the absence of saturation.

10.7717/peerj.13730/supp-2 Table S1 The mtDNA sequences of the four deep-sea echinoids

10.7717/peerj.13730/supp-3 Table S2 The species used in the phylogenetic analysis based on PCG sequences

10.7717/peerj.13730/supp-4 Table S3 The best-fit substitution model of nucleotide and amino acid substitution

10.7717/peerj.13730/supp-5 Table S4 The characteristics of the newly determined 4 echinoid mitogenomes

10.7717/peerj.13730/supp-6 Table S5 Base composition and codon usage of echinoid mitogenomes

10.7717/peerj.13730/supp-7 Table S6 The saturation of substitution for all the PCGs, PCG12 and PCG3

## Additional Information and Declarations

### Data Availability

The following information was supplied regarding data availability: The mitogenomes are available at NCBI: ON254173–ON254176. The sequences are available in the Supplementary File.
