# Supplementary figures and images for: Complete mitochondrial genomes of four deep-sea echinoids: conserved mitogenome organization and new insights into the phylogeny and evolution of Echinoidea
Source: PeerJ. 2022 Jul 28;10:e13730. doi: 10.7717/peerj.13730 (PMC9339218; doi:10.7717/peerj.13730)

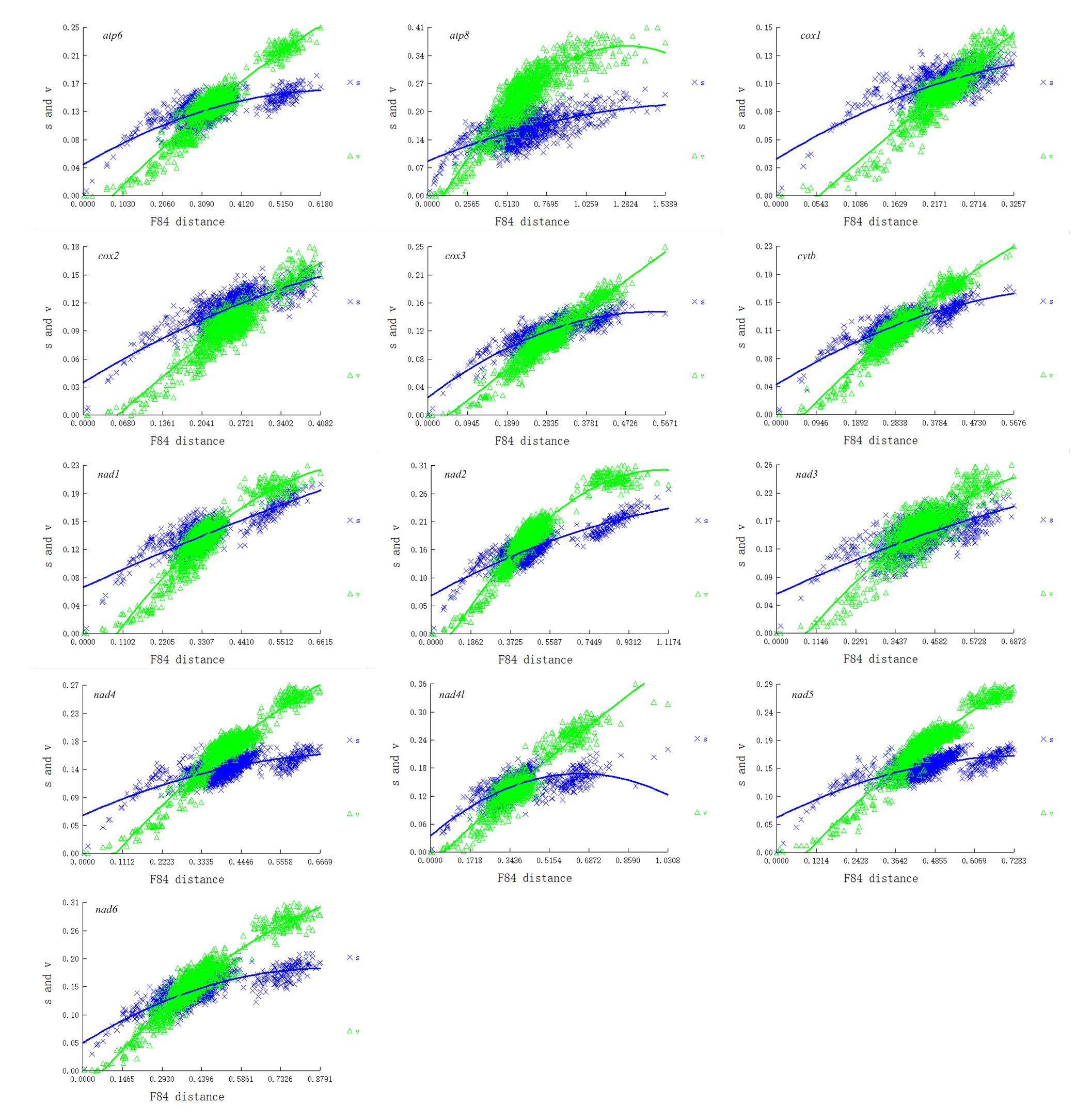

Supplement: Figure S1 — The number of transitions (s) and transversions (v) is plotted against the F84 genetic distance. A linear correlation is sustained for both transitions and transversions as expected in the absence of saturation. [file peerj-10-13730-s001.png]
